# Text Message System for the Prediction of Colonoscopy Bowel Preparation Adequacy Before Colonoscopy: An Artificial Intelligence Image Classification Algorithm Based on Images of Stool Output

**DOI:** 10.1016/j.gastha.2024.09.011

**Published:** 2024-09-19

**Authors:** Chethan Ramprasad, Divya Saini, Henry Del Carmen, Lev Krasnovsky, Rajat Chandra, Ryan Mcgregor, Russell T. Shinohara, Eric Eaton, Meghna Gummadi, Shivan Mehta, James D. Lewis

**Affiliations:** 1 Division of Gastroenterology, University of Pennsylvania, Philadelphia, Pennsylvania; 2 Perelman School of Medicine at the University of Pennsylvania, Philadelphia, Pennsylvania; 3 Perelman School of Medicine at the University of Pennsylvania, Center for Clinical Epidemiology and Biostatistics, Philadelphia, Pennsylvania; 4 Department of Computer and Information Science, University of Pennsylvania, Philadelphia, Pennsylvania

**Keywords:** Artificial Intelligence, Colonoscopy, Bowel Preparation, Technology Positive

## Abstract

**Background and Aims:**

Inadequate bowel preparation which occurs in 25% of colonoscopies is a major barrier to the effectiveness of screening for colorectal cancer. We aim to develop an artificial intelligence (machine learning) algorithm to assess photos of stool output after bowel preparation to predict inadequate bowel preparation before colonoscopy.

**Methods:**

Patients were asked to text a photo of their stool in the commode when they believed that they neared completion of their colonoscopy bowel preparation. Boston Bowel Preparation Scores of 7 and below were labeled as inadequate or fair. Boston Bowel Preparation Scores of 8 and 9 were considered good. A binary classification image-based machine learning algorithm was designed.

**Results:**

In a test set of 61 images, the binary classification machine learning algorithm was able to distinguish inadequate/fair preparation from good preparation with a positive predictive value of 78.6% and a negative predictive value of 60.8%. In a test set of 56 images, the algorithm was able to distinguish normal colonoscopy duration (<25 minutes) from long colonoscopy duration (>25 minutes) with a positive predictive value of 78.6% and a negative predictive value of 65.5%.

**Conclusion:**

Patients are willing to submit photos of their stool output during bowel preparation through text messages before colonoscopy. This machine learning algorithm demonstrates the ability to predict inadequate/fair preparation from good preparation based on image classification of stool output. It was less accurate to predict long duration of colonoscopy.

## Introduction

Colonoscopies are widely used to screen for colorectal cancer and for the diagnosis and surveillance of gastrointestinal disease.^1^ For the colon to be appropriately visualized by the endoscopist, the colon must be adequately cleansed before the procedure.^2^^,^^3^ If the colon is not adequately prepared for colonoscopy, there can be higher rates of missed pathology including colorectal cancer or precancerous lesions, complications, increased length of procedure, musculoskeletal strain to the endoscopist, and possible need for repeat colonoscopy due to inadequate inspection.^4^ The rates of inadequate bowel preparation for outpatient colonoscopy can be as high as 25%–30%.^5^

Patients are generally prescribed a fixed dose of preparation solution but the dose required for adequate preparation may vary by patient. Monitoring of stool with real-time adjustment of the bowel-cleansing regimen can improve the quality of bowel preparations. For example, for inpatient colonoscopies, patients drink preparation fluid until the stool output is clear and without solid components. This process relies heavily on the provider-dependent assessment of the stool clarity but the dose required for adequate preparation varies significantly. For outpatients, there is a lack of a validated real-time tool to predict bowel preparation adequacy during or following consumption of the purgative solution.

The use of deep learning (artificial intelligence) technology has become increasingly common in gastroenterology with automated detection of polyps during colonoscopy ^6^ as well as classification of stool caliber on the Bristol Stool Scale.^7^, ^8^, ^9^ Once trained, these algorithms can have similar or superior accuracies to trained human professionals.

We hypothesize that use of a deep learning (artificial intelligence) algorithm to predict inadequate bowel preparation could ultimately guide patients to take more preparation if indicated, allowing for real-time modification of the bowel preparation regimen before the colonoscopy. The algorithm could reduce the need for repeated procedures due to inadequate cleansing. In this cross-sectional study, we develop and assess the feasibility of a new text messaging system that includes an artificial intelligence algorithm to identify inadequate bowel preparation.

## Methods

A cross-sectional study design was utilized to develop and then assess performance of the artificial intelligence algorithm to identify inadequate bowel preparation. This quality improvement study was evaluated as exempt by the University of Pennsylvania institutional review board.

### Patient Population

All adult patients who underwent outpatient colonoscopy for any indication at Penn Presbyterian Medical Center between June 13, 2022, and August 19, 2022, were considered for this study. All patients were instructed to use a bowel preparation that included bisacodyl 20 mg and MiraLAX 238 g with clear liquid split dose bowel preparation. Patients unable to take photos with cell phone, patients, or caregivers unable to see or visualize text messages, and patients unable to provide consent or speak and read English were excluded. There was no specific camera phone requirement other than having photo-taking capability with text messaging. Patients at Penn Presbyterian Medical Center for outpatient colonoscopy are an insured patient population.

### Data Collection

Data were extracted from the electronic medical record (EPIC)© and Provation© endoscopy reporting system.

During the week before outpatient colonoscopy, patients were texted with a brief outline of the hospital initiative of submitting photos of bowel preparation before colonoscopy. All patients regardless of the time of their procedure were called at 5 pm the night before their procedure to obtain verbal informed consent. The research staff explained that the endoscopy unit is utilizing a new system to better understand and improve the bowel preparation process. Patients were asked if they have a camera phone with text message capability and if they are comfortable using it. If the patient agreed, they then received text message instructions to text a photo of their stool output the morning of their colonoscopy when they believed that they were nearing completion of their preparation, using the phrases “when you think your preparation is clear” or when “you are nearing the end of preparation related diarrhea.” Participants were sent a sample photo representing the appropriate lighting, distance, and contents of the submitted photo. There were no strict requirements for distance from the commode nor flash requirement. Any patients who did not pick up the phone were called a second time at 8 pm. Questions about the initiative, its purpose, and the process of involvement were answered over the phone, as applicable. Although it was initially considered to collect multiple photos during the bowel preparation process for each patient, patients were asked to text just 1 photo at the end of preparation for the sake of simplicity and ease. Time between text submission of image and procedure time was not collected.

After completion of the colonoscopy, the endoscopic report of adequacy of preparation using Boston Bowel Preparation Score (BBPS), duration of colonoscopy (time to cecum, procedure time), number of polyps, number of tubular adenomas were extracted from the electronic medical record. Adenoma detection rates for colonoscopy of 15% or higher for female patients and 25% or higher for male patients are indicators of adequate colonoscopy quality in the field of gastroenterology.^10^ Characteristics of age, sex, race, and indication for colonoscopy were extracted for all patients including those patients who did not submit photos via text message.

### Main Study Outcomes

The primary endpoints were adequacy of bowel preparation as measured by the BBPS and duration of colonoscopy. On the BBPS scale, a BBPS score of 5 or lower for the entire colon, or at least one segment with score of 1 or 0 is defined as inadequate preparation. BBPS of 6 or greater is considered adequate preparation.^11^ For the purposes of this study, BBPS scores of 8 or 9 were labeled as “good preparation.” BBPS scores of 7 and below were labeled as “inadequate/fair preparation.” If BBPS was not reported for the colonoscopy, the description of the preparation by endoscopist was utilized to assign the appropriate category. For example, “excellent” or “good” prep was labeled as “good preparation.” “Fair/adequate” or “inadequate/poor/unsatisfactory” was labeled as “inadequate/fair preparation.” Duration of colonoscopy was dichotomized at the 75th percentile to define longer duration. Duration of colonoscopy was chosen as a prediction target due to its importance as a marker of efficiency in endoscopy units.

### Model Design and Development

**Figure 1:**
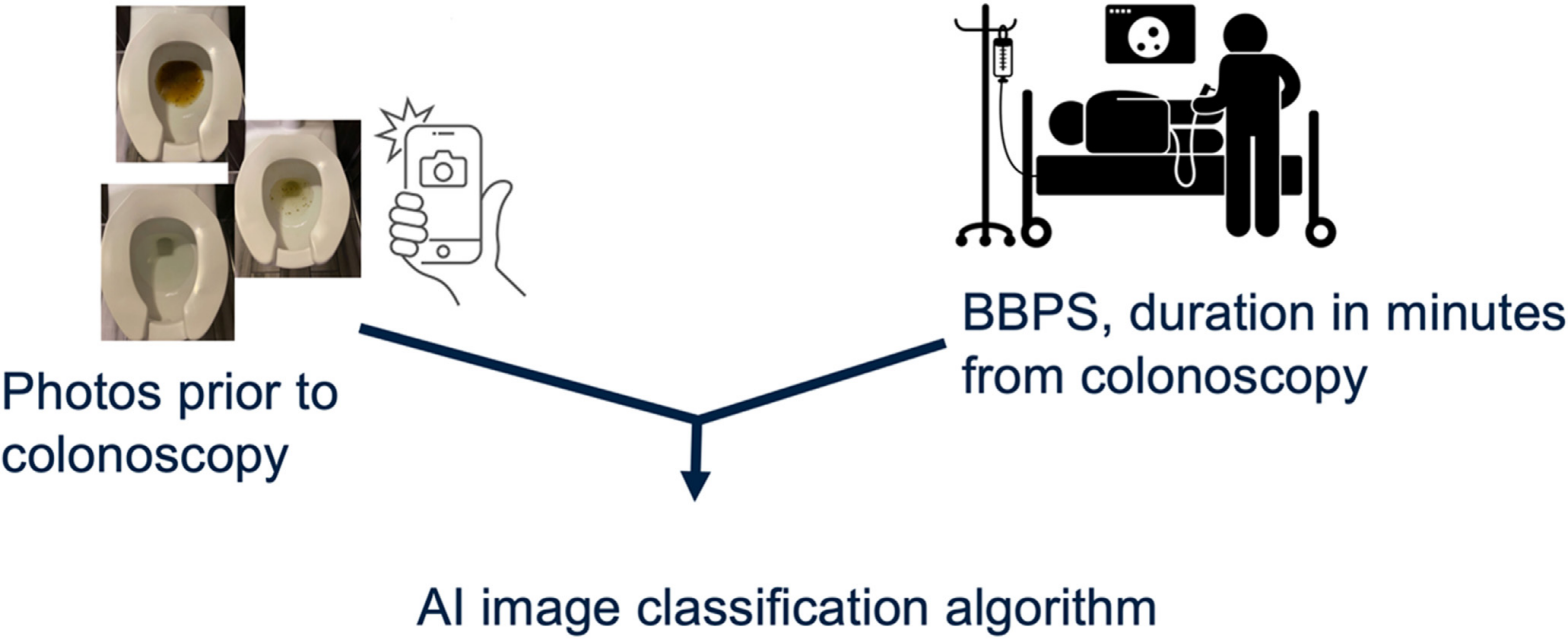
Development of artificial intelligence algorithm.

The deep learning algorithm was built by correlating images of stool output with the true findings from colonoscopy, specifically BBPS and duration of colonoscopy (Figure 1). The data were split for training, validation, and testing sets. At random, 80% of the stool images were designated for training and validation. The remaining 20% of the stool images were set aside for a test set. The algorithm was trained on 258 photos for prediction of adequacy and 219 photos for prediction of duration. The training set composition was 70.2% “good preparation” and 73.1% “normal duration of colonoscopy.” We trained the classification model using transfer learning on a pretrained ResNet-34 convolutional neural network in pyTorch. Specifically, after initializing the model’s weights via pretraining from ImageNet, we fine-tuned it end-to-end on the training images using the cross-entropy loss over the preparation quality labels to each training image of stool output. Cross-entropy loss, the widely used classification, was chosen for its stability during gradient-based optimization. The scores used by the loss allow us to assign relative probabilities to the model’s predictions. The hyperparameters were tuned using 5-fold cross validation. The Stochastic Gradient Descent optimizer was used for training the models at a learning rate of 0.001, weight decay of 0.0001, and momentum of 0.9 over 50 epochs. Given a new image of preparatory stool output from the test set, the model is then able to infer the preparation quality. Images were not preprocessed before feeding into the model. The model outputs a softmax score which we use to compute performance metrics. We report the true positive rate, false positive rate along with the f1-score, and accuracy to evaluate the performance of the models.

**Figure 2:**
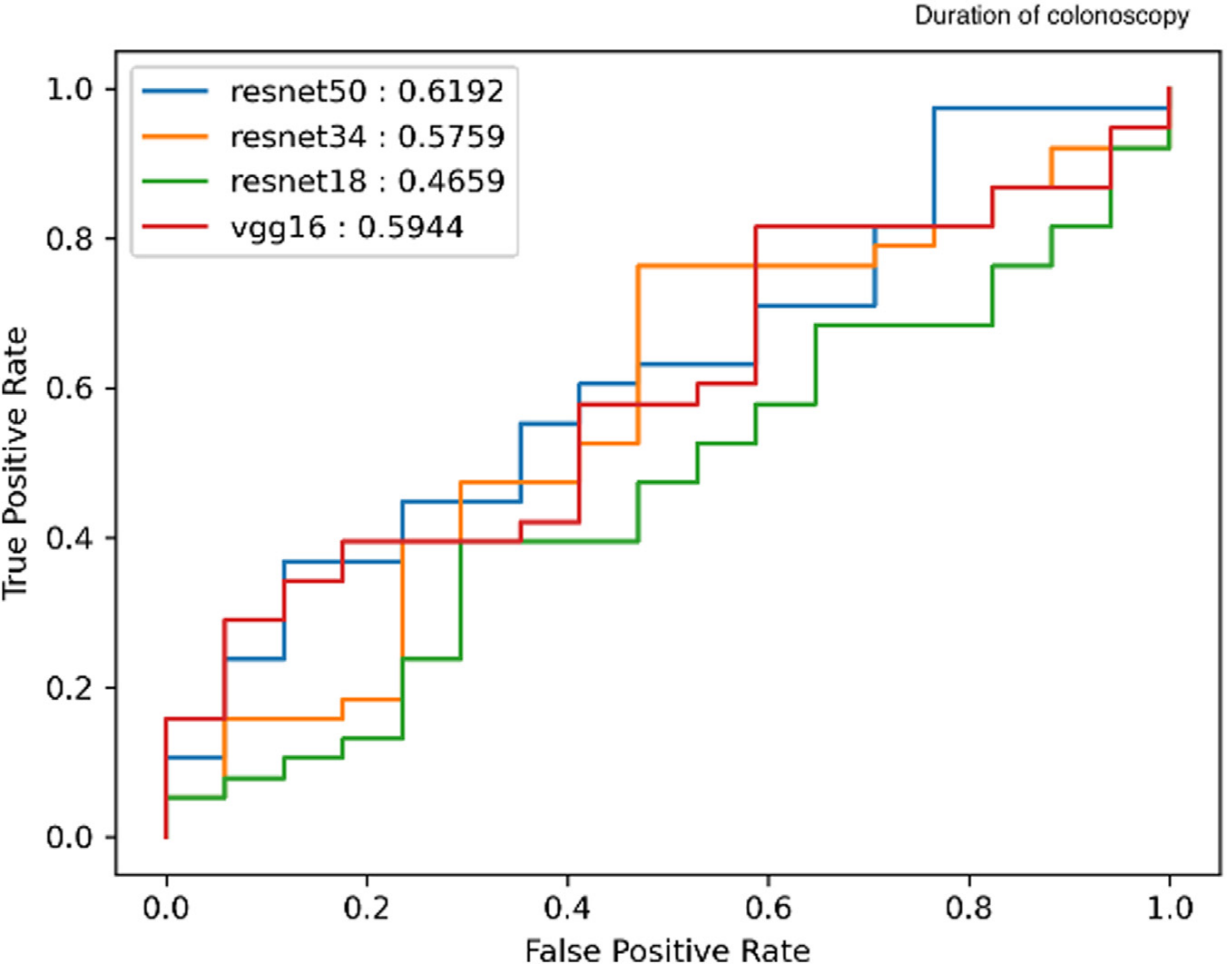
Performance analysis of alternative convolutional neural networks.

To further analyze the performance of the ResNet-34 network and why it was chosen, we evaluated the performance of alternate networks namely ResNet-50, ResNet-18, and VGG-16 for comparison. To maintain experimental consistency, each network was also pretrained on Imagenet. The receive operating characteristic curves in figure shows the discriminative ability of each binary classifier (Figure 2). From the figure we see that the ResNet-34 performs best overall. This allowed us to use the same network for both classifiers maintaining consistency.

About 20% of the stool images selected at random and the true findings from colonoscopy, specifically BBPS and duration of colonoscopy were set aside as the test set and were not included in the initial algorithm building phase. The test set included a balanced set of photos that were 57.4% “good preparation” and 57.1% “normal duration.”

### Statistical Analysis

The accuracy of the algorithm was tested on these stool images and their true findings from colonoscopy. The positive predictive value, negative predictive value, sensitivity, and specificity with 95% confidence intervals were calculated for prediction of inadequate bowel preparation.

The focus of the current study is a feasibility study for the development of a new artificial intelligence algorithm to identify inadequate bowel preparation through images of stool output before colonoscopy. Of all patients undergoing outpatient colonoscopy, the percentage of patients who submitted a text message photo was calculated. To assess factors that could impact text submission of photos as well as the model prediction performance, characteristics of age, sex, race, and indication for colonoscopy were compared between patients who submitted photos through the system and those who did not submit photos through the system. Total BBPS and duration of colonoscopy from colonoscopy findings was compared between these 2 groups. Differences were analyzed using t-test for continuous variables and Chi-squared for categorical variables. Logistic regression was used for evaluating differences in adenoma detection rate between submission groups adjusting for age, gender, and race. Difference in duration of colonoscopy was analyzed between the groups adjusting for number of polyps found during colonoscopy. Data for other factors influencing bowel preparation such as patients’ diet, medications, or comorbidities were not collected for analysis.

## Results

Data were available and extracted for 622 patients who underwent outpatient colonoscopy during the study time period. The median age was 56.7 years with standard deviation = 14.1. Of these patients, 41.2% were male and 323 (51.9%) were Black. Average risk screening colonoscopies accounted for 220 (35.7%) of all colonoscopies. Of all colonoscopies performed for which the BBPS was reported, the mean total BBPS total was 7.8 with standard deviation = 1.5. In total, there were 25 colonoscopies (4.08%) with inadequate, poor, or unsatisfactory preparation. A total of 271 (44.21%) were rated as “excellent” preparation.

**Figure 3:**
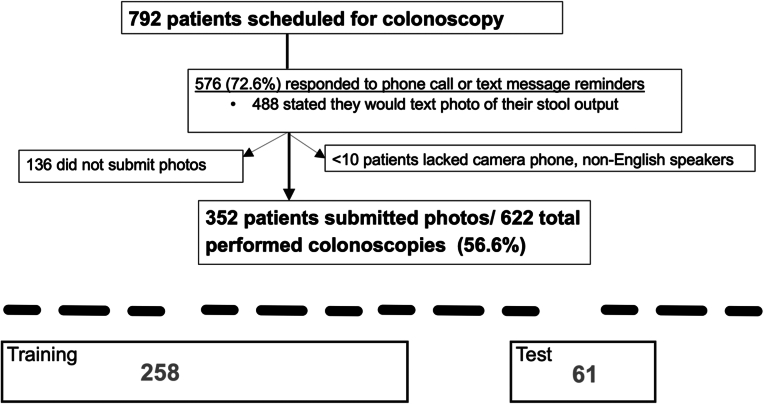
Flowchart of patients who submitted photos of stool output out of total patients scheduled for colonoscopy.

Of all 792 patients scheduled for colonoscopy during the study period, 576 (72.6%) responded to phone call or text message reminders, of which 488 (84.7%) stated that they would text a photo of their stool output. A total of 352 patients submitted photos of their stool output which is 72.1% of patients who stated they would submit a photo and 44% of all patients scheduled for colonoscopy. Ultimately, this was 56.60% (352 of 622) of all patients who had been scheduled for colonoscopy and then ultimately underwent colonoscopy (Figure 3).

Patients who submitted text message photos as compared to those who did not submit text message photos of their stool output were younger (54.6 years–59.4 years, *P* < .01, Table 1). There were no differences in sex or indication for colonoscopy. Patients who submitted photos had higher total BBPS (8.0–6.5, *P* < .01), even after adjusting for age on regression analysis (*P* < .01). Of the total 25 colonoscopies with inadequate, poor, or unsatisfactory preparation, 18 (72%) were patients who did not submit a photo. Duration of colonoscopy was shorter for those who submitted text messages (*P* = .04) but when adjusted for number of polyps, there was no significant difference (*P* = .785). The adenoma detection rate was higher for patients who did not submit photos (38.7%–28.74%, *P* = .01) but when adjusted for age, gender, and race, there was no significant difference (*P* = .74).

**Table 1:** Characteristics of Patients by Submission of Photos

Variable	Patients who submitted photos (n = 352)	Patients who did not submit photos (n = 270)	Between patients who submitted vs those you did not	All patients
Age (mean, SD)	54.6 (13.9)	59.4 (14.0)	*P* < .01	56.74 (14.1)
Sex				
% Male	144 (41.4%)	112 (40.9%)	*P* = .90	41.2
% Female	204 (58.6%)	162 (59.1%)		
Race				
Black	160 (45.98%)	163 (59.49%)	*P* = .02	323 (51.93%)
Asian	10 (2.87%)	4 (1.46%)		14 (2.25%)
White	164 (47.13%)	95 (34.67%)		259 (41.64%)
Indication (average risk screening for CRC)	129 (37.1%)	91 (33.2%)	*P* = .32	35.37
Total BBPS (mean, SD)	8 (1.4)	6.5 (1.8)	*P* < .01	7.76 (1.5)
Right BBPS	2.61 (0.6)	2.08 (0.6)	*P* < .01	2.52 (0.6)
Transverse BBPS	2.79 (0.5)	2.37 (0.7)	*P* < .01	2.73 (0.5)
Left BBPS	2.66 (0.5)	2.14 (0.7)	*P* < .01	2.57 (0.6)
Poor/fair prep	36 (10.4%)	53 (19.9%)	*P* = .02	89 (14.5%)
Excellent/good prep	310 (89.6%)	214 (80.1%)		524 (85.4%)
Duration in minutes	21.4 (8.6)	24.09 (13.3)	*P* < .01	22.5 (11.0)
Short duration <25 min	205 (69.5%)	142 (65.4%)		
Long duration >25 min	90 (30.5%)	75 (34.5%)		

CRC, colorectal cancer; SD, standard deviation.

**Figure 4:**
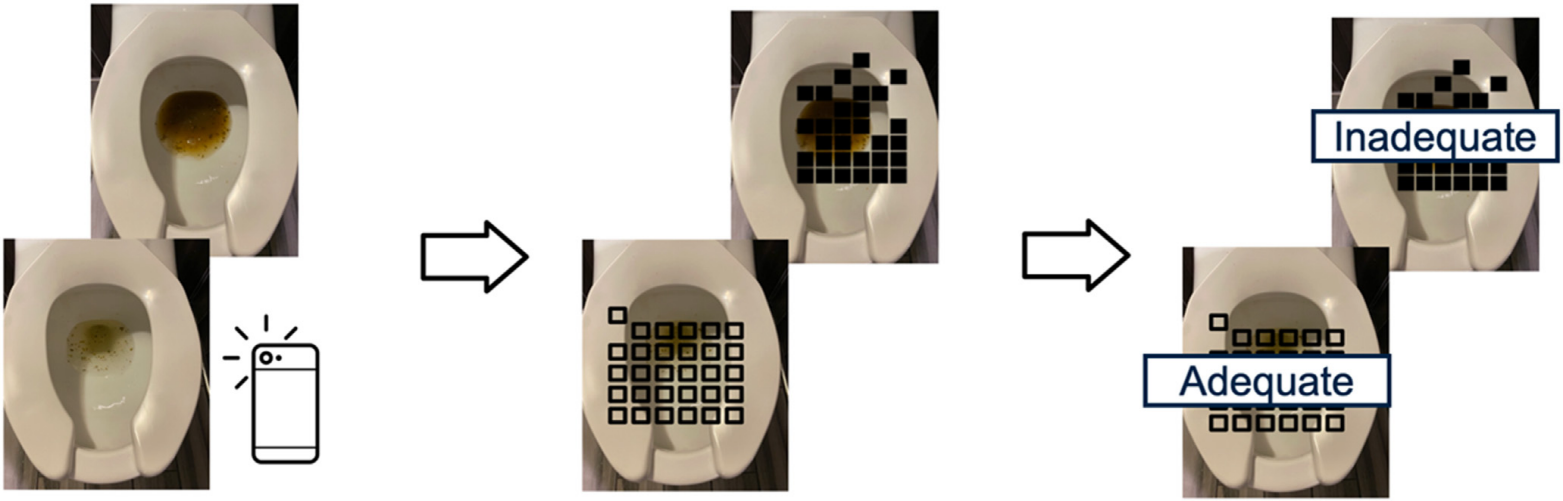
Analysis of stool output images by artificial intelligence algorithm.

**Figure 5:**
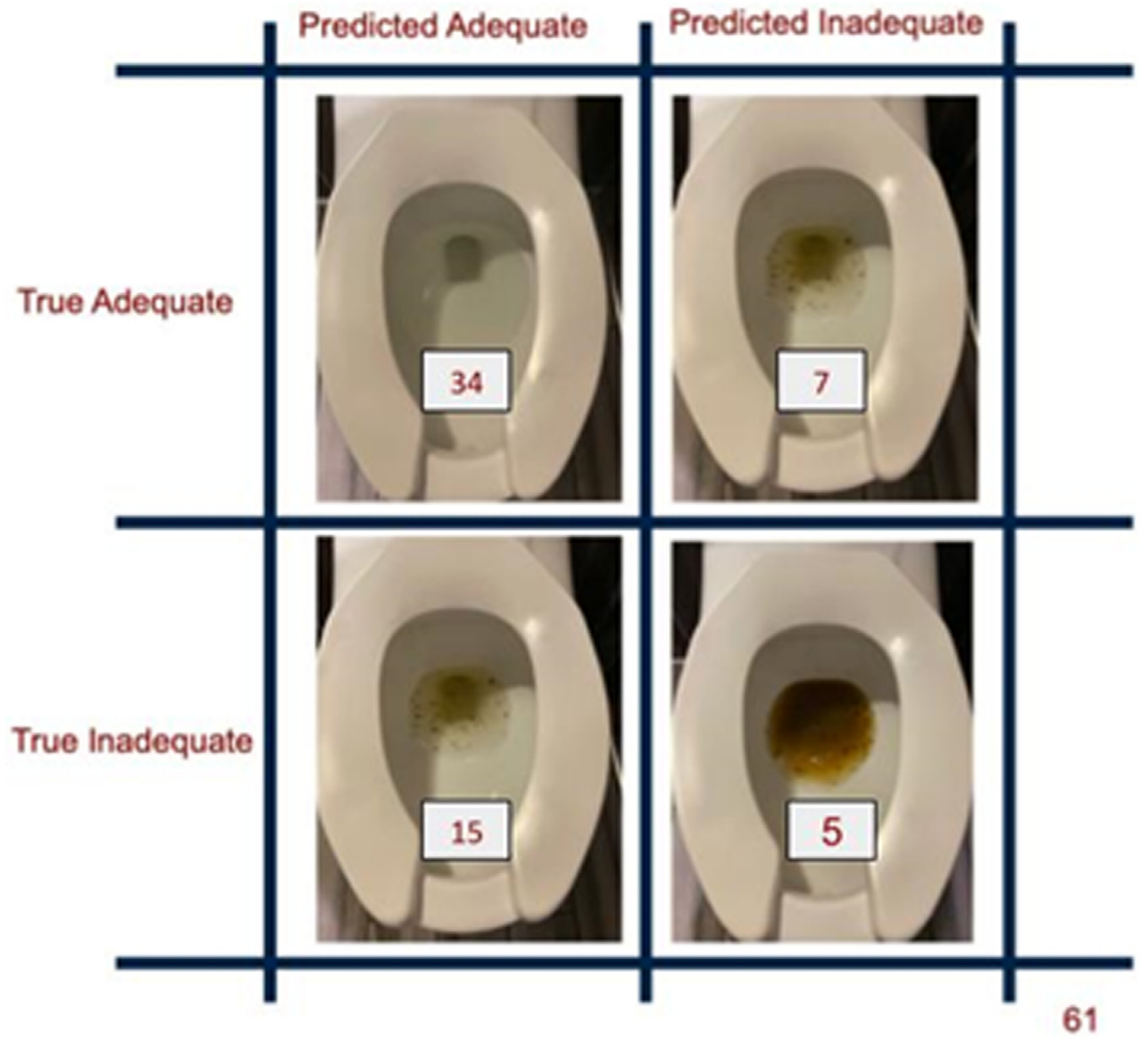
Algorithm performance for prediction of adequacy of bowel preparation.

In a test set of 61 images, the binary classification machine learning algorithm was able to distinguish inadequate/fair preparation (BBPS below 6, 6, and 7) from good preparation (BBPS scores 8 and 9) with a positive predictive value of 78.6% (95% confidence interval: 77.1%–80.0%) and a negative predictive value of 60.84% (57.92%–63.76%), sensitivity of 82.9% (78.9%–86.9%), and specificity of 53.3% (47.1%–59.6%) (Figures 4 and 5). In a test set of 56 photos, the algorithm was able to distinguish normal colonoscopy duration (<25 minutes) from long colonoscopy duration (>25 minutes) with a positive predictive value of 78.6% (75.0%–82.3%) and a negative predictive value of 65.5% (57.2%–73.9%) with sensitivity 89.5% (85.76%–93.2%) and specificity 45.1% (33.0%–57.1%) (Table 2).

**Table 2:** Accuracy of Binary Classification Machine Learning Algorithm

Variable	PPV	NPV	Sensitivity	Specificity	F1,acc
BBPS designation <6/6/7 vs 8/9	78.55%	60.84%	82.92%	53.33%	0.81, 0.73
Normal vs long duration	78.62%	65.51%	89.47%	45.10%	0.84, 0.76

NPV, negative predictive value; PPV, positive predictive value.

## Discussion

Results of this study suggest that an artificial intelligence algorithm with text messaging can be built to successfully predict inadequate bowel preparation in real time based on stool output before colonoscopy. Patients are willing and do submit photos of stool preparation through text messages. Photo submission of stool output for analysis before colonoscopy could be an effective intervention to lower the rates of inadequate bowel preparation, specifically if patients with inadequate bowel preparation are counseled appropriately to take additional preparation. Ultimately this could decrease health-care costs and improve efficiency of endoscopy units by decreasing the rates of inadequate bowel preparation.

The strengths of this study include a diverse patient population of varying age from 20 to 88 years. The population was 51.9% Black or African American, a community with historically lower access to adequate colorectal screening.^12^ The patient population also included patients undergoing colonoscopy for colorectal cancer screening or surveillance, inflammatory bowel disease surveillance, or patients with past colonic surgery as well as patients with caregivers who assisted patients with preparation. Patients in this study were requested to text photos when they felt they had completed the preparation process. Empowering the patient to understand when their stool output was nearing completion is important for further interventions to improve bowel preparation. In this time period, patients can take additional preparation before arriving to the endoscopy unit.

Differences between patients who did not submit text photos of their stool output and those who did submit photos suggest that older patients may not be as likely to comply with a text message system. Additionally, those who did not submit photos ultimately had lower total quality bowel preparation and longer duration colonoscopy. This lower total quality bowel preparation was seen even for those patients who stated that they would submit a photo but did not ultimately send a photo (mean total BBPS = 6.5). The worse quality of preparation is likely more related to medical comorbidities and social determinants of health than it is related to the actual act of submitting a text message photo. Nevertheless, understanding the differences between these groups is important for implementation of this intervention in the future. These differences affect the likelihood of submission of photos and may have impact on model prediction performance. There should be targeted focus in subsequent development of this algorithm to advocate for these populations. For example, older patients may require the assistance of a caregiver during the preparation process. Caregivers, rather than patients alone, can be instructed specifically on how to submit photos of bowel preparation. Additionally, it may be that individuals who do not follow instructions well could have poor compliance for both the bowel preparation as well as submission of photos. It may also be considered that individuals who know that they have a poor bowel preparation may not submit a photo that shows their poor preparation. Understanding these differences will be crucial for future iterations of this system to best educate and assist patients who have the worst bowel preparations.

The sample size for building this machine learning algorithm, particularly for inadequate preparation, is smaller than many other algorithms in the field of artificial intelligence. For the purposes of this study to develop the first phase of an algorithm, there is demonstrated proficiency in identifying inadequate bowel preparation. The positive predictive value, negative predictive value, and specificity are relatively high, but like most machine learning algorithms would be expected to improve with continued refinement. The accuracy of this algorithm, specifically sensitivity, is expected to increase with greater sample size for training. Additionally, this algorithm can be strengthened in the future with implementation of other image classification technology including saliency mapping to guide photo capture of the toilet bowl. Saliency mapping is a strategy utilized in image capture to guide the user to take a photo in a specific location.

The rate of inadequate, poor, and unsatisfactory preparation in this population (4.08%) is significantly lower than the published rate of 20%–30%.^2^ This may be due to the patient population, in which more than half of the colonoscopies were not average risk screening. Many of the patients may have completed a bowel preparation previously and as such may have been more compliant with preparation. It could also be due to the endoscopists’ preference for significant irrigation and cleaning the colon during colonoscopy before evaluating the BBPS score of bowel preparation. The BBPS score is not routinely reported in our practice upon entry before cleaning the colon. This is a limitation to our study design as the extent of lavage is subjective and highly variable between endoscopists. Finally, it is possible that the process of sending photos of the bowel preparation leads to greater adherence with the bowel preparation protocol as patients who submitted a photo had higher BBPS than the nonparticipants. Future studies should randomly assign patients to the intervention and correlate BBPS score before and after lavage. Additionally, future studies could assess the algorithm’s ability to differentiate poor preparation from nonpoor preparation. This was not possible in our study due to the low reported rate of inadequate preparation.

There was a small number of patients (<10) who could not participate in text submission of photos of their stool output due to the lack of a camera phone or because they were non-English speakers. In future studies in other patient populations, this could be a significant barrier to text message submission. These patients will require additional assistance. Our study did not explicitly examine attitudes of patients towards taking photographs of their stool but demonstrated a high level of participation, suggesting comfort and ease with submission of photos.

Other published research has recently looked into the use of artificial intelligence for identifying inadequate bowel preparation before colonoscopy ^13^^,^^14^ but differs significantly in methodology and approach. Lu et al. developed an algorithm that utilizes adequacy evaluation by an endoscopy nurse as the gold standard for adequacy rather than utilizing the true adequacy score of the bowel preparation from the actual colonoscopy. In contrast, our study utilized the BBPS provided by the endoscopist as the gold standard for training the algorithm. Additionally, the outcome of duration of colonoscopy in minutes is not included in the algorithm described by Lu et al. Other published work has examined the utility of artificial intelligence for intracolonoscopy evaluation based on videos of the procedure itself^15^^,^^16^ which does not predict adequacy of bowel preparation before the colonoscopy.

Patients are willing to submit photos of their stool output during colonoscopy bowel preparation through text messages. Our machine learning algorithm demonstrates the ability to predict inadequate/fair preparation from good preparation based on image classification of text message images of stool output. It was less accurate in predicting duration of colonoscopy. Identifying inadequate bowel preparation before colonoscopy creates potential to intervene in real time to lower the rates of inadequate bowel preparation and thereby improve efficiency of endoscopy units. In the long term, this could improve access to care for colorectal cancer screening and lower health-care costs.
